# Lack of Connexins 40 and 45 Reduces Local and Conducted Vasoconstrictor Responses in the Murine Afferent Arterioles

**DOI:** 10.3389/fphys.2020.00961

**Published:** 2020-08-07

**Authors:** Sophie Møller, Jens Christian Brings Jacobsen, Niels-Henrik Holstein-Rathlou, Charlotte M. Sorensen

**Affiliations:** Department of Biomedical Sciences, Faculty of Health and Medical Sciences, University of Copenhagen, Copenhagen, Denmark

**Keywords:** gap junction, vascular conducted response, electrical pulse stimulation, renal, intercellular communication

## Abstract

The juxtaglomerular apparatus (JGA) is an essential structure in the regulation of renal function. The JGA embodies two major functions: tubuloglomerular feedback (TGF) and renin secretion. TGF is one of the mechanisms mediating renal autoregulation. It is initiated by an increase in tubular NaCl concentration at the macula densa cells. This induces a local afferent arteriolar vasoconstriction and a conducted response that can be measured several 100 μm upstream from the juxtaglomerular segment. This spread of the vasomotor response into the surrounding vasculature likely plays a key role in renal autoregulation, and it requires the presence of gap junctions, intercellular pores based on connexin (Cx) proteins. Several Cx isoforms are expressed in the JGA and in the arteriolar wall. Disruption of this communication pathway is associated with reduced TGF, dysregulation of renin secretion, and hypertension. We examine if the absence of Cx40 or Cx45, expressed in the endothelial and vascular smooth muscle cells respectively, attenuates afferent arteriolar local and conducted vasoconstriction. Afferent arterioles from wildtype and Cx-deficient mice (Cx40 and Cx45) were studied using the isolated perfused juxtamedullary nephron preparation. Vasoconstriction was induced *via* electrical pulse stimulation at the glomerular entrance. Inner afferent arteriolar diameter was measured locally and upstream to evaluate conducted vasoconstriction. Electrical stimulation induced local vasoconstriction in all groups. The local vasoconstriction was significantly smaller when Cx40 was absent. The vasoconstriction decreased in magnitude with increasing distance from the stimulation site. In both Cx40 and Cx45 deficient mice, the vasoconstriction conducted a shorter distance along the vessel compared to wild-type mice. In Cx40 deficient arterioles, this may be caused by a smaller local vasoconstriction. Collectively, these findings imply that Cx40 and Cx45 are central for normal vascular reactivity and, therefore, likely play a key role in TGF-induced regulation of afferent arteriolar resistance.

## Introduction

The juxtaglomerular apparatus (JGA) is a specialized structure, formed by the afferent and efferent arteriole, thick limb of the ascending loop of Henle, and mesangial cells. The JGA embodies two major renal functions: tubuloglomerular feedback (TGF), which is part of the mechanism underlying renal autoregulation, and renin secretion. The JGA consists of different cell types with specialized functions: the macula densa (MD) cells, juxtaglomerular cells, mesangial cells, vascular smooth muscle cells (VSMCs), and endothelial cells (ECs). The MD cell of the thick limb of the ascending loop of Henle functions as a NaCl sensor. An increase in NaCl concentration in the tubular fluid eventually causes the VSMC in the afferent arteriole to contract to reduce glomerular filtration rate (GFR) and the juxtaglomerular cells of the afferent arteriole to reduce renin secretion. Mesangial cells are located between the MD cells (the sensor) and the afferent arteriole (the effector). They are believed to play an important role in the signal transduction from the MD to the afferent arteriole (Goligorsky et al., 1997).

The TGF signal originates in the MD cells and elicits local vasoconstriction in the juxtaglomerular part of the afferent arteriole. The response, however, encompasses the entire afferent arteriole, the distal part of the interlobular artery, and travels into the afferent arterioles of neighboring nephrons (Peti-Peterdi, 2006; Marsh et al., 2009). This indicates that the vasoconstrictor signal is conducted within the vascular wall most likely through gap junctions (GJs) coupling ECs to ECs, VSMCs to VSMCs, and ECs to VSMCs.

GJs are fluid-filled pores that allow the movement of small molecules and current between neighboring cells. GJs are made up of two hemichannels, connexons, built from connexins (Cxs) (Nielsen et al., 2012). Six Cxs of the same or different isoforms can assemble to form a connexon, and the two connexons from neighboring cells can dock to form a functional GJ channel (Moreno, 2004). Several Cx isoforms are expressed in the JGA (Hanner et al., 2010). Similarly, in preglomerular arterioles, expression of Cx37, Cx40, Cx43, and Cx45 (Wagner, 2008; Just et al., 2009; Schweda et al., 2009) is found, enabling the cells of the vessel wall, both smooth muscle and endothelial, to act as a syncytium (Goligorsky et al., 1997; Dora et al., 2003; Haefliger et al., 2004; Peti-Peterdi, 2006; Yao et al., 2009).

In the vasculature, Cx40 is the most widely expressed isoform and is predominantly found in the ECs (de Wit et al., 2003; Schweda et al., 2009), as well as in renin-producing cells (Haefliger et al., 2001). In mice, deletion of Cx40 leads to the development of renin-dependent hypertension (de Wit et al., 2000, 2003; Figueroa et al., 2003; Wagner et al., 2007; Just et al., 2009; Sorensen et al., 2012) and reduced conduction of vasodilation (de Wit et al., 2000). Cx45 is expressed in the VSMCs (Wagner, 2008) and mesangial cells (Kurtz et al., 2009; Hanner et al., 2010), and lack of Cx45 expression causes lethal malformations in the vasculature (Krüger et al., 2000; Hanner et al., 2008; Wagner, 2008). Therefore, only a conditional deletion of Cx45 is possible in mice. Previous studies in these mice show that Cx45 plays a role in the propagation of VSMC calcium waves and in TGF (Hanner et al., 2008; Møller et al., 2020). This calcium signaling may be one mechanism by which Cx45 affects vascular conducted responses.

Once the signal from MD has reached the afferent arteriole, the vascular conduction can spread dilation or constriction along the vessel wall, depending on the blood flow needs (Wagner, 2008). Conducted vasoconstriction occurs when depolarizing current propagates upstream *via* GJs (Gustafsson and Holstein-Rathlou, 1999), from the afferent arterioles to the larger arteries, and changes the vascular resistance, thus reducing renal blood flow (Wagner et al., 1997).

In this study, we aim to test if a lack of two specific Cxs affects vascular conduction in the afferent arteriole. Specifically, we explore the roles of Cx40 and Cx45 in the conduction of vasoconstrictor responses to electrical pulse stimulation.

## Materials and Methods

### Animal Preparation

Procedures were approved by the Danish National Animal Experiments Inspectorate. All animals were kept in the animal facility at University of Copenhagen and received tap water and standard chow *ad libitum*. Genotyping was done on DNA from ear clippings using the DirectPCR kit (Viagen Biotech; Los Angeles, CA).

Cx40 wildtype (Cx40 WT) and Cx40 knockout (Cx40 KO) mice were on a mixed C57Bl/6 × 129S4/SvJae genetic background. Mice were purchased for breeding from the European Mouse Mutant Archive (infrafrontier.eu, Munich, Germany). Kidneys from a total of 15 adult mice were used for the studies (Cx40 WT *n* = 8, five females and three males and Cx40 KO *n* = 7, four females and three males). The age of the mice ranged from 4 to 14 months.

Homozygous female mice with floxed Cx45 gene (Cx45^fl/fl^, >87% C57Bl/6 background) were mated with homozygous Cx45^fl/fl:Nestin-Cre^ males, >87% C57Bl/6 background. Breeding pairs were a generous gift from Dr. Klaus Willecke, University of Bonn, Germany; the mice have been described previously (Maxeiner et al., 2005). In their offspring, the Cx45 coding DNA is replaced with an enhanced green fluorescence protein (eGFP) in cells expressing the intermediate filament Nestin during development. The Cx45^fl/fl^ mice are phenotypically wildtype and are used as such (WT). The Cx45^fl/fl:Nestin-Cre^ mice are used as our knockout (KO). Kidneys from 20 adult mice were used for the studies (Cx45 WT *n* = 9, three females and six males and Cx45 KO *n* = 11, nine females and two males), ranging from 2 to 6 months of age.

### Imaging System and Software

**Figure 1:**
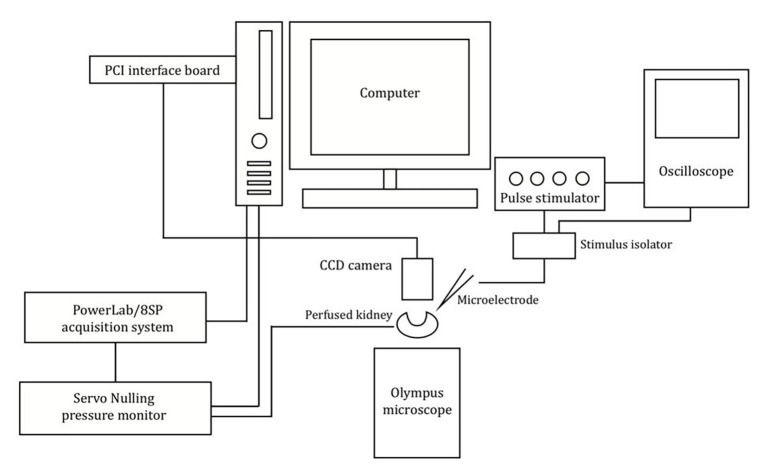
Schematic of the imaging system and experimental setup used. Schematic showing the setup used to measure the afferent arteriolar diameter in response to electrical stimulation.

The experimental setup is shown in Figure 1. The vasculature was viewed and recorded using an Olympus BX50WI microscope with a digital 12-bit CCD camera (Pixelfly, PCO, Kelheim, Germany) mounted on the microscope. Images were recorded using the CamWare software (PCO, Kelheim, Germany). Renal perfusion pressure (RPP) readings were obtained using the PowerLab/8SP data acquisition system (ADInstruments, Colorado Springs, CO) and viewed using LabChart 7 software (ADInstruments, Colorado Springs, CO). Post-experimental evaluations of afferent arteriolar diameter were performed offline using ImageJ (National Institute of Health, Bethesda, MD).

### Surgical Procedure

Experiments were performed using the *in vitro* perfused juxtamedullary nephron preparation, developed by Casellas and Navar (1984), and adapted to mice (Harrison-Bernard et al., 2003; Sorensen et al., 2012). Mice were anesthetized with pentobarbital (50 g/kg *i.p.*, Mebumal, SAD, Denmark). The abdominal aorta was catheterized and immediately perfused with Tyrode’s solution (in mmol/L: 136.9 NaCl, 0.42 NaH_2_PO_4_, 11.9 NaHCO_3_, 2.7 KCl, 2.2 MgCl_2_, 5.6 d-glucose, and 1.8 CaCl_2_) containing 5% BSA (ICPbio International, Auckland, New Zealand) and an amino acid mixture (in mmol/L: 2 alanine, 2 glycine, 5 glutamine, 1 serine, and supplemented with MEM amino acid solution, all from Sigma-Aldrich, Copenhagen, Denmark) at pH 7.4. Kidneys were excised, and the cannula was advanced into the renal artery of the left kidney. The kidney was decapsulated and sectioned longitudinally exposing the intact papilla, which was reflected revealing the inner cortical surface. Venous tissue on the cortical surface was cut, allowing access to the arterial vasculature. Perfusion was isolated to the inner cortical vessels by ligating larger arterial vessels (Dafilon 10/0 sutures, B. Braun Vet Care GmbH, Tuttlingen, Germany).

The cannula system comprised of a 27-gauge blunt needle and a polyethylene (PE-10) line, connected to a servo-nulling pressure system (Instrumentation for Physiology and Medicine, San Diego, CA), allowing for continuous RPP monitoring. RPP was maintained at 95 mmHg throughout the experiment by adjusting the regulator, controlling the flow of 95% O_2_-5% CO_2_ gas mixture to the perfusion solution reservoir.

### Experimental Procedure: Application of Electrical Pulse Stimulation

**Figure 2:**
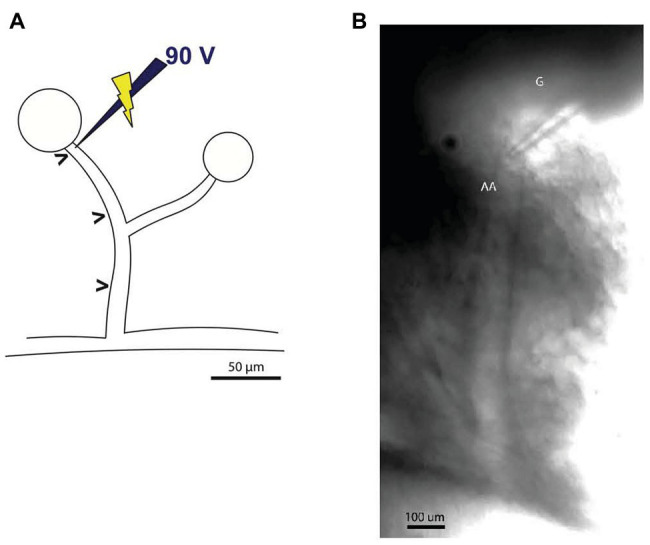
Illustration of the microelectrode positioning for electrical pulse stimulation. **(A)** Stimulation microelectrode was positioned at the glomerular entrance of the afferent arteriole. The diameter was measured at sites labeled (>), corresponding to the stimulation site and sites (50 μm apart) upstream from the microelectrode (maximum distance reached only in some experiments were 450 μm). Schematic representation inspired by Wagner et al. (1997). **(B)** Image of the experimental setup showing the stimulation electrode placed immediately upstream from glomerulus (G) just above the afferent arteriole (AA).

Borosilicate glass micropipettes (CMA Microdialysis, Kista, Sweden) were pulled on a horizontal Flaming/Brown micropipette puller (Model P-87, Sutter Instrument Co., Novato, CA) and back-filled with a 2 mol/L NaCl solution containing Lissamine green (Sigma-Aldrich, Copenhagen, Denmark). The microelectrode was connected to a stimulus isolator (ISO-flex, A.M.P.I., Jerusalem, Israel) connected to a stimulator (Model S44, Grass Instrument Co., Quincy, MA) and an oscilloscope (1200 A, Hewlett-Packard, Palo Alto, CA). The microelectrode (0.5–0.8 MΩ resistance) was placed at the glomerular entrance of the afferent arteriole, where local afferent vasoconstriction was induced by electrical pulse stimulation (2.5 Hz frequency, 300 ms pulse duration, and 90 V amplitude; Salomonsson et al., 2002; Palanker et al., 2008), as shown in Figure 2. A wire was placed in the tissue chamber to serve as the grounding electrode. After a recovery period, an experimental protocol consisting of 30 s recordings of baseline, electrical pulse stimulation, and recovery periods was initiated. The vessels were stimulated for 30 s, a period which was sufficient for it to reach a new steady-state diameter (Steinhausen et al., 1997).

### Diameter Measurements

**Figure 3:**
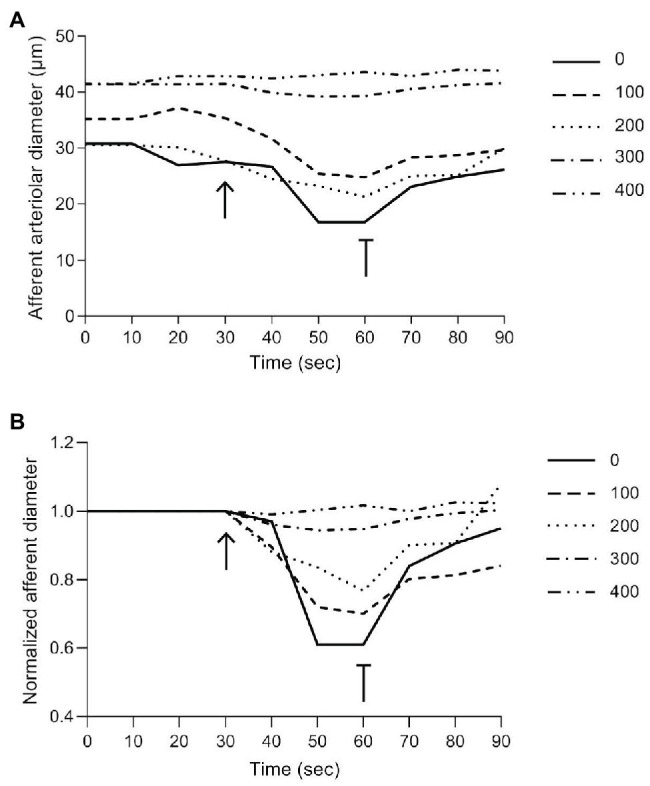
Representative trace of a typical experimental recording from Cx40 WT. **(A)** Absolute changes in afferent arteriolar diameter as a function of time at the stimulation site (0) and at increasing distances from the stimulation site (100–400). **(B)** Changes in normalized afferent arteriolar diameter as a function of time (diameter was normalized by the corresponding local mean diameter prior to the electrical stimulation). The solid line (0) represents the diameter measurements at the stimulation site, while dotted/dashed lines represent upstream measurements. The arrow and T-bar indicate when electrical stimulation was initiated and stopped.

The afferent arteriolar diameter was measured by tracking the vessel edges and the inner diameter was measured every 10 s throughout the experiment. The diameter was measured at the stimulation point at the glomerular entrance (local response) as well as every 50 μm upstream from the glomerulus (conducted response; Figures 2, 3) as far as it was possible to track the vessel (Table 1). Each data point represents the average of three consecutive measurements (after 10, 20, and 30 s of stimulation) in the same position.

**Table 1:** Mean values of body weight prior to kidney removal, age, pre-stimulated vessel diameter and traceable vessel length starting at the stimulation point on the afferent arteriole.

		N	Body wt. (g)	Age (weeks)	AA diameter (μm)	Measured vessel length (μm)
Cx40	WT	8	33.1 ± 1.3	31.8 ± 5.6	28.7 ± 5.3	259.4 ± 58.2
	KO	7	27.9 ± 1.7	41.9 ± 4.1	15.5 ± 3.4	178.6 ± 26.4
			*p* = 0.04	NS	*p* = 0.04	NS
Cx45	WT	9	27.4 ± 1.3^*^	16.4 ± 1.1^***^	30.6 ± 2.7	361.1 ± 28.6
	KO	11	22.5 ± 1.6^#^	15.9 ± 1.2^###^	23.6 ± 3.1	295.5 ± 41.8
			*p* = 0.007	NS	NS	NS

Cx40, connexin 40; Cx45, connexin 45; WT, wildtype; KO, knockout; AA, afferent arteriole; NS, not significant.

* *p* < 0.05;

*** *p* < 0.001 vs. Cx40 WT;

# *p* < 0.05;

### *p* < 0.001 vs. Cx40 KO.

Experiments were included if they fulfilled the following: (1) electrical stimulation induced a minimum of 5% constriction in the vessel diameter (local response), (2) post-stimulation, the vessel recovered to at least 50% of its baseline diameter, and (3) diameters were measurable at a minimum of three positions along the vessel (including stimulation site).

### Data Handling and Statistical Analyses

Graphical presentation and statistical analyses were performed using SigmaPlot (Systat Software Inc. San Jose, CA, USA), MatLab (MathWorks, Natick, MA, USA), and Rstudio (version 1.3.959; Boston, MA). Vessel diameter during stimulation was normalized to the respective mean resting diameter at the given distance from the stimulation site. Normalized and pooled data for contraction at the stimulation site (0 μm) from each experimental series were evaluated using a one-way ANOVA to test for differences between WT and KO. A one-sample *t*-test testing against 0 was used to evaluate significant constriction at each measurement point.

**Figure 4:**
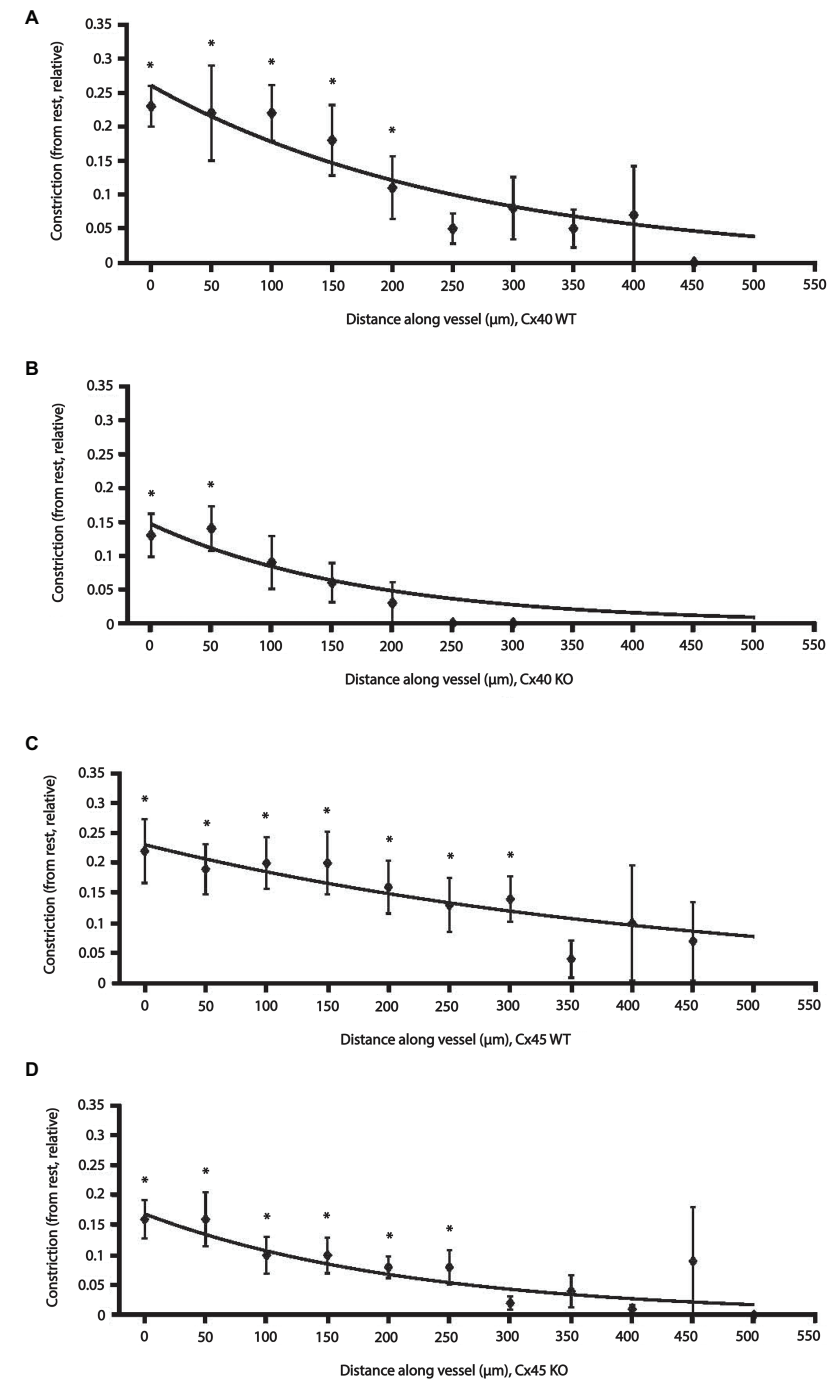
Effect of electrical stimulation on afferent arteriolar diameter in WT and KO of Cx40 and Cx45 mice. Changes in afferent arteriolar diameter as a function of distance from the stimulation point. Data are constriction relative to the local diameter measured at rest. Data are presented as mean ± SEM. The lines are fits to the data points weighted according to the number of observations at each distance and fitted to an exponential model: y = ae^bx^ (please see Methods and Materials section). Data points without error bars: *n* = 1. **(A)** Cx40 WT (*n* = 8) and **(B)** Cx40 KO (*n* = 7). **(C)** Cx45 WT (*n* = 9) and **(D)** Cx45 KO (*n* = 11). ^*^ *p* < 0.05 vs. 0.

Length constants of the conducted vasoconstriction were calculated in MATLAB® (The MathWorks Inc., R2017b) for each type of animal by a non-linear least squares fit of the data to the equation *y* = *ae^bx^*, where *y* is the change in diameter at upstream distance *x* from the stimulation site, *a* is the *y*-intercept, and *b* is the decay rate of the response along the vessel. When conducted vasoconstriction reached 0 or a negative value, constriction was set to 0 for the remaining measurements. The maximum distance from the stimulation point at which the individual vessel could be visualized varied from 100 to 500 μm. Consequently, there were more measurements at the shorter distances. All measurements at a given distance were pooled for each group. Therefore, in each fit, the shorter distances with more measurements weighs in more heavily than longer distances with fewer measurements (Figure 4; constants shown in Table 2). Each group gives rise to a single fit. To enable statistical comparison of the decay rates between groups, a boot strapping procedure was applied (source code available in Supplementary Data). In brief, for each group of animals, a set of data points were obtained by drawing randomly, with replacement, from the original data set. The single exponential decay model was then fitted to this new data set to obtain estimates of *a* and *b*. The procedure was repeated 1,000 times, giving new sets of *y*-interceptions and decay rates. Differences in the latter between WT and KO groups were analyzed using a two-tailed *t*-test.

**Table 2:** Summary of conducted response characteristics for WT and KO animals of both strains.

Strain	a, 95% CI (μm)	b (×10^−3^), 95% CI (×10^−3^) (1/μm)	Length constant (−1/b, μm)
Cx40 WT	0.26, (0.20, 0.33)^*^	−3.8, (−6.0, −1.7)	363
Cx40 KO	0.15, (0.092, 0.20)	−5.6, (−10.7, −0.47)	179
Cx45 WT	0.23, (0.17, 0.29)	−2.2, (−3.8, −0.53)^**^	455
Cx45 KO	0.17, (0.12, 0.21)	−4.5, (−7.1, −2.0)	222

Values are calculated by fitting data to a single exponential decaying function:$y=ae^{\mathrm{bx}}$ *y*=aebx where *y* is the relative change in diameter at distance *x* upstream from the stimulation site, *a* is the *y*-intercept, and *b* is the decay rate of the response along the vessel. Significance levels were evaluated using bootstrapping procedure.

* *p* < 0.01 Cx40 WT vs. Cx40 KO;

** *p* < 0.05 Cx45 WT vs. Cx45 KO.

Previous studies have shown no difference in the measured renal vascular responses between male and female mice, and data were therefore pooled for statistical comparison (Boesen et al., 2012; Brasen et al., 2018; Gohar et al., 2019; Møller et al., 2020). *p* ≤ 0.05 was considered statistically significant. All values are reported as means ± SEM unless otherwise stated.

## Results

### Animals

Mean values of body weight, age, pre-stimulated vessel diameter (measured at 95 mmHg), and traceable vessel length are summarized in Table 1. Body weights were significantly higher in Cx40 WT mice (33.1 ± 1.3 g) compared to Cx40 KO mice (27.9 ± 1.7 g, *p* = 0.04) and in Cx45 WT mice (27.4 ± 1.3 g) compared to Cx45 KO mice (22.5 ± 1.6 g, *p* = 0.007). Mice from the Cx40 strain were significantly heavier than the Cx45 strain. Age was not different within the groups; however, mice from the Cx40 strain were older than the mice from the Cx45 strain. Resting vessel diameter measured at the glomerular entrance was significantly different between Cx40 WT and Cx40 KO (*p* = 0.04), but not between Cx45 WT and Cx45 KO, or between groups. Maximal traceable vessel length (afferent arteriole and in some cases interlobular arteries) was not significantly different between or within groups.

### Afferent Arteriolar Diameter

Figure 2 shows an afferent arteriole branching from an interlobular artery and the micropipette used to deliver the electrical pulse stimulation.

A typical recording of the afferent arteriolar diameter from a Cx40 WT mouse is shown in Figure 3A and the normalized diameter is shown in Figure 3B. The electrical stimulation (shown by the arrow at 30 s) produces strong vasoconstriction locally (0 μm). Vessel diameter is reduced by approximately 40% of its baseline diameter within the first 20 s of stimulation. Termination of the electrical stimulation (shown by the T-bar at 60 s) results in a quick return to near control diameters. Locally, diameter returned to approximately 85% of its baseline diameter within the first 10 s and by the end of the 30-s recovery, the diameter has returned to near baseline value. The magnitude of the vasoconstriction response is seen to decrease with increasing distances upstream from the stimulation site. At 400 μm, electrical stimulation induced no detectable vasoconstriction in the example shown.

Figure 4 shows the relative reduction in vessel diameter as a function of distance from the stimulation site in Cx40 WT and KO (Figures 4A,B). Figures 4C,D show data from Cx45 WT and KO, respectively. A data point (♦) in any of the figures represents the average of all the measurements made at that given distance ± SEM. The exponential curve fit is weighted according to the number of observations at a given distance (source code available in Supplementary Data). Thus, data points close to the stimulation site with many observations weigh in more heavily than data points further away with fewer observations. Data for the fitted curves are shown in Table 2.

Comparing measurements at the stimulation site directly between groups (one-way ANOVA) showed that relative vasoconstriction between Cx40 WT and KO (0.23 ± 0.03 vs. 0.13 ± 0.03; *p* < 0.05) was significantly different. Albeit numerically different, no significant difference was found between Cx45 WT and KO (0.22 ± 0.05 vs. 0.16 ± 0.03; *p* = 0.32). This is in line with the fitted data (Table 2), where the *y*-intersection (*a*) is equivalent to the local vasoconstriction.

The decay rate (*b*) of the conducted vasoconstriction evaluated using bootstrapping was significantly smaller in Cx45KO compared to Cx45 WT (*p* < 0.05, Table 2) corresponding to a larger length constant in the Cx45WT. There was no significant difference in decay rate between Cx40WT and Cx40KO although it appeared smaller in Cx40 KO.

In all groups, vasoconstriction decreases with increasing distance. Comparing the relative afferent arteriolar constriction in each group against 0, the data showed that in Cx40 WT a significant constriction was measurable up to 200 μm (Figure 4A), in Cx40 KO the distance was 50 μm (Figure 4B). In the Cx45 group, a significant constriction could be measured at 300 μm in the WT arterioles (Figure 4C) and at 250 μm in the KO (Figure 4D).

## Discussion

The present study investigated the importance of intercellular communication *via* GJs, through Cx40 and Cx45, in the conduction of afferent arteriolar vasoconstriction in response to local electrical stimulation. We have previously shown that lack of Cx40 or Cx45 significantly reduces TGF (Sorensen et al., 2012; Møller et al., 2020). Here, we examine if this reduction is caused by a decrease in the vascular conduction of vasoconstriction. The local and conducted vasoconstrictor responses were measured during stimulation and compared to the diameter measured before stimulation. Decay of constriction was computed for the stimulation period for data pooled from each mouse strain.

VSMCs and ECs work together to coordinate and propagate constriction and dilation along the vessel wall (Li et al., 2015). The conducted vasomotor response is important in the regulation of blood flow and resistance in vascular networks (Gustafsson et al., 2001) and depends on GJs (Segal et al., 1989; Hakim et al., 2008; Braunstein et al., 2009). In afferent arterioles, ECs are primarily coupled through Cx40 (Arensbak et al., 2001) and VSMCs through Cx45 (Møller et al., 2020). GJs also play a role in the signaling between ECs and VSMCs (Emerson and Segal, 2000) through myoendothelial GJs (MEGJs; Haefliger et al., 2004). It is unknown which Cxs are part of MEGJs in the renal vasculature (Wagner, 2008) but Cx45 or Cx40 are likely candidates. In Cx40 KO mice, renal endothelium derived hyperpolarization was significantly reduced compared to WT, suggesting that Cx40 is part of the renal MEGJ (Brasen et al., 2018). Also, Cx40 has been found in junctions between EC and renin secreting cells (transformed VSMC; Haefliger et al., 2001) supporting that Cx40 could be part of the MEGJ.

Both mouse strains used in the present study have been characterized previously. The Cx40 KO was hypertensive (de Wit et al., 2000; Wagner, 2008; Just et al., 2009), but RBF was similar in Cx40 WT and KO (Just et al., 2009). Segmental arterial and afferent arteriolar diameters were not significantly different (Sorensen et al., 2012), but heart weight and afferent wall trans-sectional area were increased in Cx40 KO mice (Sorensen et al., 2012; Jacobsen and Sorensen, 2015), possibly due to the hypertension. The Cx45 KO was reported to be slightly hypertensive (Hanner et al., 2008) or normotensive (Schmidt et al., 2012). Segmental arterial and afferent arteriolar diameters were not significantly different between Cx45 WT and KO, but wall trans-sectional area was decreased in afferent arterioles from Cx45 KO (Møller et al., 2020). In both strains, TGF was found to be reduced in the KO (Sorensen et al., 2012; Møller et al., 2020), whereas the myogenic response was unaffected by the reduction in intercellular communication (Jacobsen and Sorensen, 2015; Møller et al., 2020).

Electrical pulse stimulation induced a local and a conducted vasoconstriction response in afferent arterioles from Cx40 and Cx45 WT and KO mice. The local response measured at the stimulation site was significantly smaller in Cx40 KO compared to Cx40 WT. However, in cremaster arterioles from Cx40 KO mice, local vasoconstriction elicited with electrical stimulation or KCl was similar to that observed in WT mice (Figueroa et al., 2003; Wölfle et al., 2007). As shown before, the afferent arteriolar diameter measured in the juxtaglomerular segment in Cx40 KO was smaller than in WT and smaller than the diameter measured 100 μm upstream (Sorensen et al., 2012). This could suggest an increased baseline afferent arteriolar constriction at the site closest to the glomerulus when Cx40 is not present. Eliminating TGF did not normalize afferent arteriolar diameter, suggesting that TGF is not causing the constriction. Importantly, afferent arteriolar diameter measured upstream from the juxtaglomerular site was not significantly smaller in Cx40 KO (Sorensen et al., 2012). No significant difference in the local vasoconstriction was found between Cx45 WT and KO even though it appeared smaller in Cx45 KO. However, this could be due to the relatively small effects being measured resulting in low statistical power. In mice lacking Cx45 in VSMC, KCl-induced local vasoconstriction was similar to the constriction elicited in WT mice (Schmidt et al., 2012). In isolated segmental arteries from both strains, no difference was found between WT and KO when assessing the vasoconstrictor response to NE (Brasen et al., 2018; Møller et al., 2020). Collectively, these data suggest that the renal vascular reactivity in Cx40 and Cx45 KO is not reduced. Possibly, the decreased juxtaglomerular afferent arteriolar diameter in Cx40 KO affects the local vasoconstriction elicited at this site.

Although the decay rate of vasoconstriction seemed slightly increased in Cx40 KO, no significant difference was found between Cx40 WT and KO. This is in accordance with results obtained in skeletal muscle arterioles, where KCl-induced conducted vasoconstriction was unaffected in Cx40 KO mice (de Wit et al., 2000). It is generally believed that electrical signals travel predominantly in the endothelial layer (Kapela et al., 2018), which is coupled mainly by Cx40. In isolated preglomerular arteries from Cx40 KO, electrical stimulation did not elicit an increase in VSMC intracellular Ca^2+^ measured 500 μm from the stimulation site (Sorensen et al., 2012). However, no measurements were made at shorter distances. In addition, an electrical signal may travel further than what can be measured when using increases in intracellular Ca^2+^ or vasoconstriction as parameters. Comparing the constriction at a given distance against 0 revealed that in Cx40 KO the constriction was only significant at 50 μm. This could be due to the smaller local constriction.

In contract, in Cx45 KO, the decay rate was significantly increased compared to WT. Possibly, Cx45 is part of the MEGJ in the afferent arteriolar wall, and its removal by conditional knockout could reduce communication between the VSMC layer and the endothelium, hence reducing the mechanical response from the VSMC layer. Myoendothelial junctions are unevenly distributed along the wall. The spread of depolarization from VSMCs that are coupled to the endothelium, to VSMCs that are not, could be central for a distant mechanical response to electrical stimulation. In addition, although the endothelium is normally considered the main path for a longitudinal spread of current in the microcirculation, the presence of VSMC-VSMC coupling by Cx45-containing GJs may also contribute. A possible proposed mechanism is through Ca^2+^ signaling as Cx45 is known to be involved in the propagation of Ca^2+^ waves in VSMC isolated from afferent arterioles (Hanner et al., 2008). Comparing the constriction against 0 showed that in Cx45 KO the constriction was only significant up to 250 μm compared to 300 μm in the WT.

The structure of the vascular wall may affect both the local vasoconstriction and conduction properties. The vascular wall in renal vessels with a diameter <40 μm is thicker compared to vessels from skeletal muscles (Hosoyamada et al., 2015), which would reduce conduction in afferent arterioles. In both KO strains, changes in wall trans-sectional area (WTA) and stress sensitivity in the afferent arteriole have been found (Jacobsen and Sorensen, 2015; Møller et al., 2020). However, WTA and stress sensitivity seemed to be increased in Cx40 KO, whereas it was decreased in Cx45 KO. Whether this can account for the increase in decay rate observed in Cx45 KO remains to be established. Another possibility is that deletion of Cx40 or Cx45 changes the expression of other GJs or ion channels responsible for eliciting the vasoconstriction. Deletion of Cx40 reduced expression of Cx37 in the vasculature (Simon and McWhorter, 2003; de Wit, 2010), which could affect the vascular response. Whether this could also be the case for vascular ion channels is currently not known.

Differences in myogenic activity at the chosen RPP of 95 mmHg may also influence the obtained results. Resting membrane potential of VSMCs in the afferent arteriole in isolated perfused rat kidneys was approximately −40 mV at 80 mmHg RPP (Loutzenhiser et al., 1997). We assume that a comparable resting membrane potential is present in mouse afferent arterioles at an RPP of 95 mmHg. Under these conditions, the afferent arteriole has substantial myogenic tone and addition of nifedipine, an inhibitor of L-type Ca^2+^ channels, significantly increased afferent arteriolar diameter (Sorensen et al., 2012). In Cx40 KO mice, afferent arteriolar diameter remained constant even though RPP was increased from 75 to 95 mmHg (Sorensen et al., 2012) and in Cx45 KO mice the same RPP increase induced a significant reduction in afferent arteriolar diameter (Møller et al., 2020), suggesting that both strains have myogenic tone in the present experimental setup. As no differences have been found in the myogenic response between KO and WT from both strains (Jacobsen and Sorensen, 2015; Møller et al., 2020) it does not seem likely that differences in myogenic tone are affecting the electrically induced vascular responses observed in the present experiments.

A number of limitations are present in our experiments. Due to the experimental setup, it is not possible to position the stimulation pipette next to the vascular wall. A varying layer of tubular tissue will separate the pipette tip and the vascular wall. This limitation has been mitigated by only including arterioles with a minimum local constriction of 5%. In addition, the preparation is not fixed and the arterioles have some vasomotion that could affect the measurements. Consequently, we used an average of the diameter measured over 30 s. Vasoconstriction is not the most sensitive measure to quantify vascular conduction. Changes in membrane potential can be measured using sharp electrodes or patch-clamp (Li et al., 2004). This method is not applicable in our preparation, where the cells of the vascular wall are not freely accessible. Another factor that should be taken into consideration is the age span of the mice (2–14 months). Laboratory mice reach sexual maturity at 8–12 weeks of age (Dutta and Sengupta, 2016); past the age of 18 months, these mice are considered old (Hagan, 2017). We know that age does not affect the myogenic response (Lott et al., 2004), unless subjects are sedentary (Hong and Lee, 2017). In WKY and SHR rats, both cortical and juxtamedullary autoregulatory activity were similar between ages 10 and 70 weeks (Iversen et al., 1998) even though MAP increased significantly in SHR. These findings indicate that vascular function is intact at 14 months of age, and we are not aware of any study that has directly addressed the effect of aging on vascular conduction in the mouse afferent arteriole. Lastly, we have used mixed sexes. Renal autoregulation in the normal physiological state is identical in males and females (Hilliard et al., 2011; Brown et al., 2012) as is renal vascular responses to angiotensin II and PE (Schneider et al., 2010). Thus, the use of mixed sexes would likely not affect the results.

In conclusion, we have shown that knockout of Cx40 reduces local vasoconstriction but does not significantly change the conduction of vasoconstriction. Vascular deletion of Cx45 significantly reduces the vascular conduction induced by electrical stimulation in afferent arterioles. Consequently, vasoconstriction in response to a comparable stimulus is conveyed shorter when intercellular communication between cells in the vascular wall is reduced in the renal microcirculation. This may in turn affect the ability to regulate and autoregulate renal perfusion.

## Data Availability Statement

All datasets generated and source code for this study are included in the article/Supplementary Material.

## Ethics Statement

The animal study was reviewed and approved by the Danish National Animal Experiments Inspectorate, Ministry of Environment and Food of Denmark, and Danish Veterinary and Food Administration.

## Author Contributions

SM and CS designed the animal experiments. SM performed animal experiments and drafted manuscript. SM, CS, and JJ analyzed data and prepared figures and tables. SM, JJ, N-HH-R, and CS interpreted results, edited and revised manuscript and approved final version of manuscript.

### Conflict of Interest

The authors declare that the research was conducted in the absence of any commercial or financial relationships that could be construed as a potential conflict of interest.
